# Emodin ameliorates bleomycin-induced pulmonary fibrosis in rats by suppressing epithelial-mesenchymal transition and fibroblast activation

**DOI:** 10.1038/srep35696

**Published:** 2016-10-24

**Authors:** Ruijuan Guan, Xia Wang, Xiaomei Zhao, Nana Song, Jimin Zhu, Jijiang Wang, Jin Wang, Chunmei Xia, Yonghua Chen, Danian Zhu, Linlin Shen

**Affiliations:** 1 Department of Physiology and Pathophysiology, School of Basic Medical Sciences, Fudan University, Shanghai, China; 2 Division of Nephrology, Zhongshan Hospital, Fudan University, Shanghai, China; 3 Department of Gastroenterology, Zhongshan Hospital, Fudan University, Shanghai, China; 4 Shanghai Key Laboratory of Medical Imaging Computing and Computer Assisted Intervention, Fudan University, Shanghai, China

## Abstract

Aberrant activation of TGF-β1 is frequently encountered and promotes epithelial-mesenchymal transition (EMT) and fibroblast activation in pulmonary fibrosis. The present study investigated whether emodin mediates its effect via suppressing TGF-β1-induced EMT and fibroblast activation in bleomycin (BLM)-induced pulmonary fibrosis in rats. Here, we found that emodin induced apoptosis and inhibited cellular proliferation, migration and differentiation in TGF-β1-stimulated human embryonic lung fibroblasts (HELFs). Emodin suppressed TGF-β1-induced EMT in a dose- and time-dependent manner in alveolar epithelial A549 cells. Emodin also inhibited TGF-β1-induced Smad2, Smad3 and Erk1/2 activation, suggesting that Smad2/3 and Erk1/2 inactivation mediated the emodin-induced effects on TGF-β1-induced EMT. Additionally, we provided *in vivo* evidence suggesting that emodin apparently alleviated BLM-induced pulmonary fibrosis and improved pulmonary function by inhibiting TGF-β1 signaling and subsequently repressing EMT, fibroblast activation and extracellular matrix (ECM) deposition. Taken together, our data suggest that emodin mediates its effects mainly via inhibition of EMT and fibroblast activation and thus has a potential for the treatment of pulmonary fibrosis.

Idiopathic pulmonary fibrosis (IPF) is a chronic, progressive, and devastating lung disease with unknown etiology and poor prognosis^1^. Most of the patients with IPF develop honeycomb lung and die of irreversible loss of pulmonary function^1^. Despite transient clinical stability, progressive deterioration is inevitable and the survival time of patients starting from the onset of symptoms is probably 3 to 5 years^2^,^3^. However, current treatment options, including pirfenidone and nintedanib (BIBF 1120), the only two drugs approved by the United States Food and Drug Administration (FDA), are able to slow the progression of IPF but not reverse the established pulmonary fibrosis^4^. IPF still remains a major leading cause of death worldwide and represents a global challenge. Therefore, it is crucial to understand the pathogenesis and explore novel therapies with improved efficacy.

IPF is characterized by the formation of fibroblast and myofibroblast foci, which secrete excessive extracellular matrix (ECM), primarily fibrillary collagens, resulting in scarring and destruction of lung architecture^5^. In this process, resident fibroblasts, the key players in fibrotic tissue remodeling, play a pivotal role. Their migration, proliferation and differentiation make great contributions to the formation of fibroblast and myofibroblast foci^6^. Further, alveolar epithelial cells (AECs) also play a role via epithelial-mesenchymal transition (EMT)^7^. Previous studies suggest a close association between the progression of IPF and EMT, via loss of epithelial markers such as E-cadherin and expression of mesenchymal markers such as fibronectin and vimentin, indicating the potential role of EMT in IPF development^8^,^9^.

Transforming growth factor (TGF)-β1, a key fibrogenic cytokine, regulates diverse cellular functions including migration, proliferation, differentiation, and apoptosis^10^,^11^. It is aberrantly expressed in the lungs of patients suffering from IPF^12^. In addition to triggering the differentiation of fibroblasts into myofibroblasts and maintenance of contractile phenotype, it also induces EMT in AECs and plays a critical role in EMT-mediated organ fibrosis via canonical and non-canonical Smad signaling^5^,^11^,^13^. Both the pathways suppress the expression of E-cadherin by zinc-finger transcription factors such as Slug, ZEB and Snail^14^. Thus, anti-EMT approach or inhibition of TGF-β1 signaling is also considered a promising therapeutic target in IPF^15^.

Emodin (1,3,8-trihydroxy-6-methylanthraquinone), an anthraquinone isolated from Rheum palmatum L. (Polygonaceae) exhibits antiviral, anti-inflammatory, anticancer, immunosuppressive and pro-apoptotic activities^16^. Recently, emodin has been reported to suppress proliferation by inducing apoptosis via the caspase-dependent pathway and exerted EMT-suppressing effect^17^,^18^,^19^. Emodin was also documented to alleviate pancreatic and hepatic fibrosis by inhibiting critical TGF-β1 protein^20^,^21^. Furthermore, emodin has been demonstrated to alleviate BLM-induced pulmonary fibrosis in mice^22^. However, the antifibrotic mechanisms have not yet been clarified. The present study was designed to investigate whether emodin mediates its antifibrotic effects through suppressing EMT and fibroblast activation by blocking activation of TGF-β1 pathway. We found that emodin induced apoptosis and inhibited cell proliferation, migration and differentiation in TGF-β1-stimulated human embryonic lungfibroblasts (HELFs). Emodin inhibited TGF-β1-induced EMT in alveolar epithelial A549 cells by inhibiting TGF-β1 signaling pathway. We also present *in vivo* evidence showing that emodin significantly repressed TGF-β1 signaling, EMT, fibroblast activation and eventual ECM deposition in the BLM-treated lungs indicating potential therapeutic efficacy of emodin in pulmonary fibrosis.

## Results

### Emodin alleviates BLM-induced pulmonary fibrosis in rats

**Figure 1:**
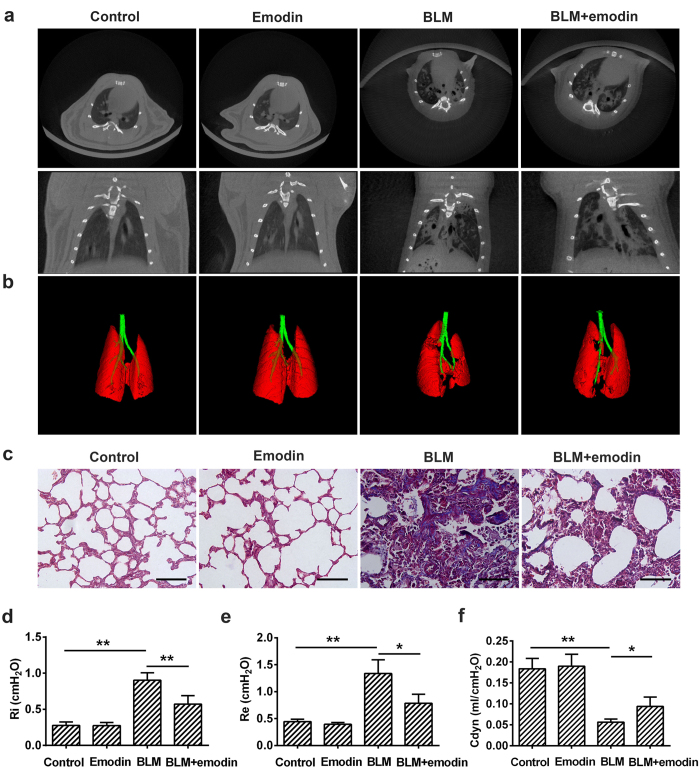
Emodin alleviates bleomycin (BLM)-induced pulmonary fibrosis in rats. Rats were intratracheally injected with a single dose of BLM (3.0 mg/kg) and subsequently received emodin (20 mg/kg) or vehicle by gavage daily for 21 days. (**a**) Representative images from different groups determined by Micro-CT showing radiologic features were presented. (**b**) Representative mimetic diagram drawn out from Micro-CT images, based on different tissues with varying density. (**c**) Lung tissue sections were prepared and stained with Masson’s trichrome staining (200x magnification). (**d–f**) Pulmonary function parameters including inspiratory resistance (Ri), expiratory resistance (Re) and pulmonary dynamic compliance (Cdyn) among different groups were compared three weeks after BLM instillation. Data are expressed as mean ± SD, n = 4, *P < 0.05; **P < 0.01.

In order to evaluate the anti-fibrotic potential of emodin *in vivo*, rats stimulated with BLM (3.0 mg/kg) as described previously^23^ were treated with 20 mg/kg emodin (daily, i.g.) for three weeks. Emodin was well tolerated by rats without any symptoms of weight loss, or acute or delayed toxicity, suggesting the selection of appropriate dosage. Interestingly, as illustrated by micro-CT of lung tissues, intratracheal injection of BLM induced the development of pulmonary parenchyma lesion with ground-glass opacities and honeycombing. Treatment of emodin ameliorated BLM-induced pulmonary fibrosis (Fig. 1a,b). Similarly, emodin intervention significantly reduced the damage to normal lung architecture and excessive deposition of collage fibers due to BLM, as determined by Masson’ trichrome staining (Fig. 1c). Further, we measured the changes in pulmonary function after emodin treatment. Impressively, the pulmonary function of emodin-treated rats was significantly improved, as seen by decreased inspiratory resistance (Ri), expiratory resistance (Re) and increased dynamic compliance (Cdyn), when compared with BLM-treated rats (Fig. 1d–f). Emodin in non-BLM-treated rats generated no changes in lung pathology or pulmonary function.

### Emodin inhibits TGF-β1-induced cell proliferation, migration and differentiation in human embryonic lung fibroblasts

**Figure 2:**
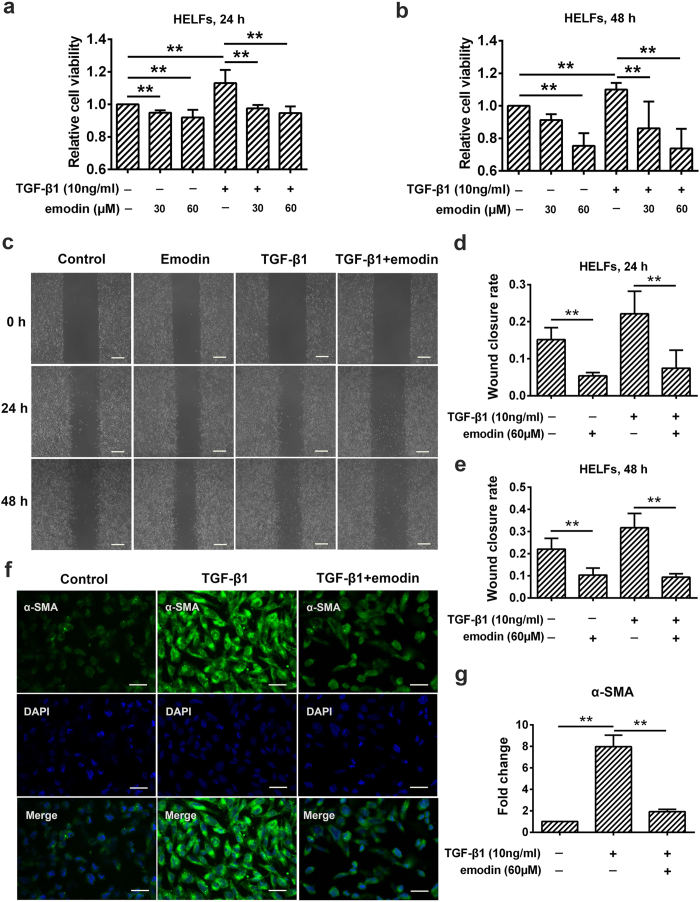
Emodin inhibits cell proliferation, migration and differentiation in human embryonic lung fibroblasts (HELFs). HELFs were treated with TGF-β1 (10 ng/mL) and emodin for 24 and 48 h, respectively. The cells treated with DMSO only served as control. (**a,b**) The viabilities of fibroblasts treated as indicated were measured by CCK-8 assay at 24 and 48 h, respectively. All the values were normalized to the control, representing 100% cell viability. (**c**) Wound healing assay was used to assess the effect of emodin on fibroblasts migration. The wound closure was photographed at post-scratching 24 and 48 h. Original magnification, ×400. (**d,e**) The wound closure rate at 24 and 48 h, representing the migration rate, was detected. (**f**) Immunofluorescence staining of α-SMA (a marker of myofibroblast differentiation) was performed. Original magnification, ×400. (**g**) Immunofluorescence of α-SMA was quantified by Image J software. Data are expressed as mean ± SD, n = 6, *P < 0.05; **P < 0.01.

We explored the detailed anti-fibrotic mechanisms of emodin. Migration and proliferation of fibroblasts/activated fibroblasts occurs at the initial stage of tissue repair in response to injury and contributes to the development of lung fibrosis^24^. To investigate the effects of emodin on the proliferation and migration of fibroblasts/activated fibroblasts, human embryonic lung fibroblasts (HELFs) were selected and cultured with and without 10 ng/mL TGF-β1 and/or 30 or 60 μM emodin for 24 and 48 h. We first explored whether emodin affected fibroblast proliferation and activation in HELFs using the CCK-8 assay. As shown in Fig. 2a,b, treatment with TGF-β1 led to an increased number of viable fibroblasts, whereas emodin significantly reduced the cell viability caused by TGF-β1 in a dose- and time-dependent manner. Emodin also reduced cell viability in a dose- and time-dependent manner in unstimulated HELFs, indicating that emodin inhibits both constitutive and inducible fibroblast proliferation. Meanwhile, would healing was also evaluated to analyze the effects of emodin on the migration of fibroblast/activated fibroblasts. As shown in Fig. 2c–e, the migration rates of both basal and TGF-β1-stimulated fibroblasts were blocked by emodin at 60 μM after 24 h of assay, suggesting the inhibitory effect of emodin on fibroblast migration. Moreover, in injured lung tissues, fibroblasts are also activated and differentiate into myofibroblasts, which are a type of specialized contractile cells with higher profibrotic potential than fibroblasts^5^. Therefore, we also investigated the effect of emodin on TGF-β1-induced myofibroblast differentiation. Immunofluorescence staining showed that TGF-β1 substantially enhanced α-smooth muscle actin (α-SMA) expression, a marker of myofibroblast differentiation, as compared to control cells. Nonetheless, emodin treatment significantly reduced TGF-β1-induced expression of α-SMA, when compared with TGF-β1-treated cells, indicating that emodin significantly inhibited TGF-β1-induced myofibroblast differentiation (Fig. 2f,g).

### Emodin promotes cell apoptosis in both basal and TGF-β1-stimulated human fibroblasts

**Figure 3:**
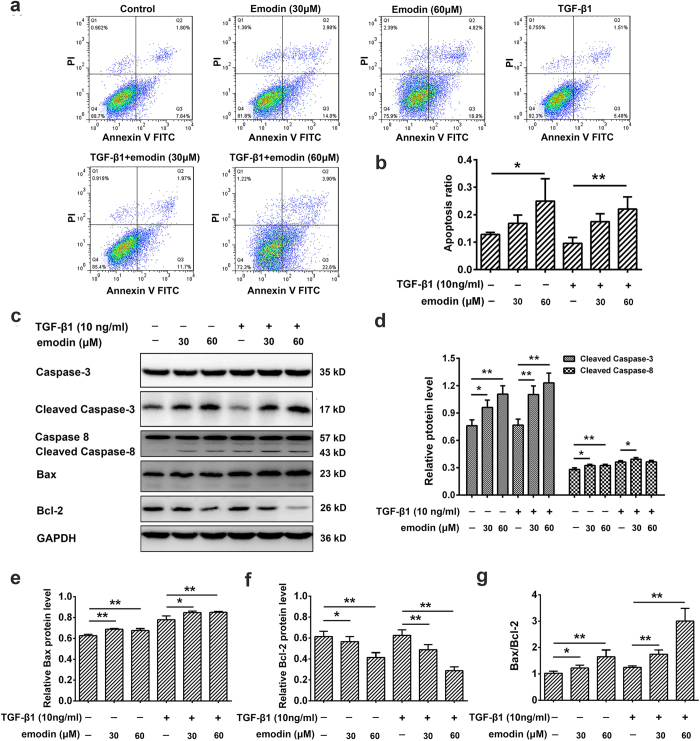
Emodin promotes cell apoptosis in both basal and TGF-β1-stimulated human fibroblasts. HELFs were treated with TGF-β1 (10 ng/mL) and the indicated concentrations of emodin (30 and 60 μM) for 24 h. (**a**) The cells were double-stained with Annexin V-FITC and PI, and then the cellular apoptosis was determined by flow cytometry. (**b**) The ratio of apoptotic cells (Annexin V+ PI-, Annexin V- PI+ and Annexin V+ PI+) was statistically analyzed. (**c**) The protein expression of caspase-3, cleaved caspase-3, caspase-8, cleaved caspase-8, Bax and Bcl-2 was analyzed by Western blot assay. (**d–f**) Densitometric analysis of proteins of interest in the immunoblots using GAPDH as the internal reference. (**g**) The ratio of Bax/Bcl-2. Data are expressed as mean ± SD; n = 6, *P < 0.05; **P < 0.01.

We used flow cytometry to determine whether the anti-proliferative effect of emodin on HELFs was mediated via apoptosis. The data revealed that treatment with emodin increased cellular apoptosis both in unstimulated and TGF-β1-stimulated fibroblasts (Fig. 3a,b). Because apoptosis is triggered mainly via caspase-mediated signal transduction, we then investigated whether emodin induced apoptosis via caspase pathway. Western blot analysis showed that emodin significantly enhanced the expression of cleaved caspase-3 and cleaved caspase-8 in both unstimulated HELFs and TGF-β1-stimulated HELFs (Fig. 3c,d). Additionally, treatment with emodin decreased the expression of anti-apoptotic protein Bcl-2 and increased the level of pro-apoptotic protein Bax (Fig. 3c,e,f). Therefore, the Bax/Bcl-2 ratio was significantly increased after emodin intervention (Fig. 3g). These results showed that emodin promotes apoptosis of fibroblasts as well as TGF-β1-stimulated fibroblasts, indicating that apoptosis might be an important mechanism underlying the anti-proliferative effect.

### Emodin represses TGF-β1-induced EMT in alveolar epithelial A549 cells

**Figure 4:**
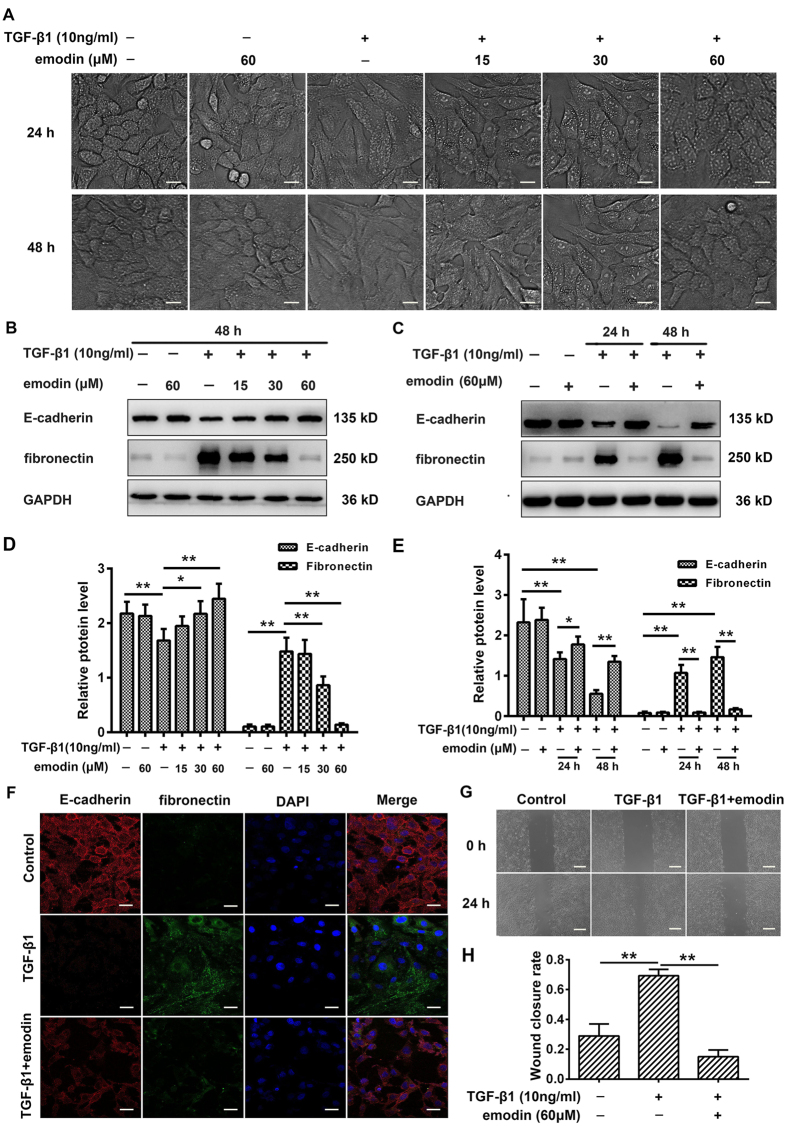
Emodin suppresses TGF-β1-induced EMT in alveolar epithelial A549 cells. A549 cells were treated with TGF-β1 (10 ng/mL) and different concentrations of emodin (15, 30, 60 μM) for 24 or 48 h. The cells stimulated with DMSO only served as controls. EMT was determined by examining (**A**) the morphological changes. Original magnification, ×400. (**B,C**) the expression levels of marker proteins of EMT including E-cadherin and fibronectin. (**D,E**) Quantitative densitometry of different samples using Western blot. Expression of E-cadherin and fibronectin was normalized to GAPDH level in the same sample. A549 cells were treated with TGF-β1 (10 ng/mL) and emodin (60 μM) for 24 h, and EMT was further examined by (**F**) immunofluorescence and (**G**) wound healing assay. (**H**) The wound closure rate, representing the migration rate, was detected. Data are expressed as mean ± SD, n = 6, *P < 0.05; **P < 0.01.

Since EMT plays a critical role in the pathogenesis of fibrosis in different tissues and organs^25^, we investigated whether the anti-fibrotic effect of emodin was partially mediated via inhibition of EMT. TGF-β1 is the principal cytokine in the induction of EMT in a wide variety of biological systems, including AECs^26^. We, therefore, initially assessed whether emodin inhibited TGF-β1-induced EMT in A549 cells (an alveolar type-II epithelial cell line). TGF-β1-induced EMT was identified by the morphological alterations ranging from an epithelial shape to a typical spindle-liked fibroblastic shape, which were associated with decreased expression of E-cadherin and increased expression of fibronectin. As expected, emodin intervention inhibited TGF-β1-induced EMT in a dose- and time-dependent manner, as determined by the changes in cellular morphology (Fig. 4A) and expression of EMT markers (Fig. 4B–E). Likewise, our immunofluorescence study further demonstrated that TGF-β1 significantly down regulated E-cadherin expression and upregulated fibronectin expression, when compared with control cells. Nevertheless, emodin treatment enhanced the expression of E-cadherin and reduced the expression of fibronectin, when compared with TGF-β1 alone treated cells (Fig. 4F). Additionally, emodin decreased the migration of AECs induced by TGF-β1, further indicating the inhibitory effect of emodin in EMT (Fig. 4G,H).

### Emodin targets TGF-β1 signaling pathway to suppress EMT

**Figure 5:**
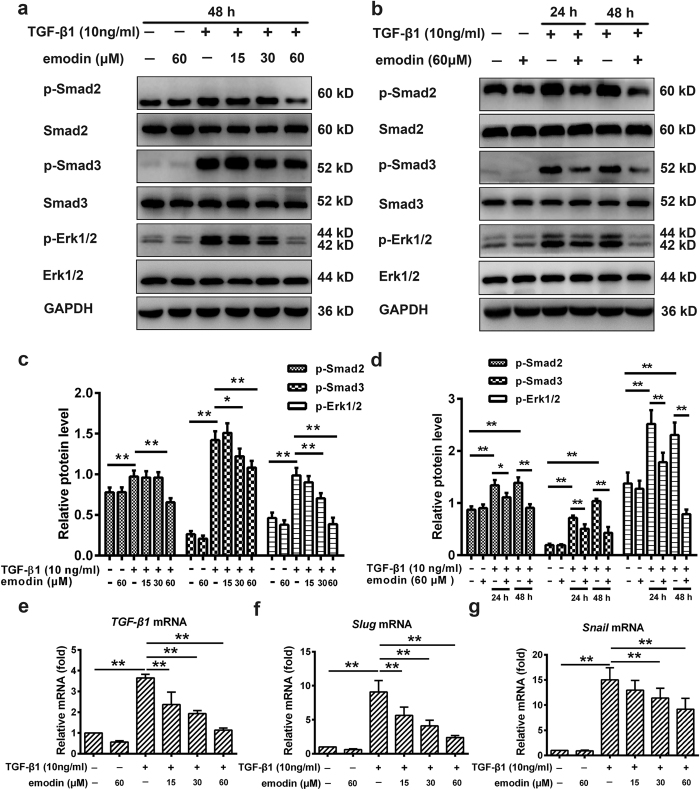
Emodin inhibits TGF-β1 signaling pathway in alveolar epithelial A549 cells. A549 cells were treated as indicated for 24 or 48 h. (**a,b**) Western blot was used to analyze the phosphorylation levels of Smad2, Smad3 and Erk1/2. Results are representative of different experiments. (**c,d**) Scanning densitometry of western blot on different samples was analyzed quantitatively. Expression of p-Smad2, p-Smad3 and p-Erk1/2 was normalized to Smad2, Smad3 and Erk1/2 level, respectively. (**e–g**) Real-time PCR was performed to examine the mRNA level of TGF-β1, Slug and Snail. Data are expressed as mean ± SD, n = 6, *P < 0.05; **P < 0.01.

The ability of TGF-β1 signaling to stimulate EMT during pulmonary fibrosis is well-documented^10^. TGF-β1 primarily activates the signal transducer Smads and Smad-mediated transcription of target genes to induce EMT^27^. Thus, we first investigated the effects of emodin on the activation of canonical Smad-dependent pathway. As shown in Fig. 5, TGF-β1-treated cells exhibited increased phosphorylation of Smad2 and Smad3, when compared with control cells. However, emodin treatment apparently reduced the phosphorylation of Smad2 and Smad3. In addition, TGF-β1 also activates MAP kinase signaling^28^. Therefore, we examined the role of emodin in the kinase cascade and found that emodin inhibited the phosphorylation of p44/42 MAPK (Erk1/2) in A549 cells, suggesting that emodin antagonized TGF-β1-mediated Smad and non-Smad signaling to inhibit EMT. Interestingly, emodin dose-dependently reduced the level of endogenous TGF-β1 transcripts (Fig. 5e). Furthermore, since Snail and Slug are recognized as the main transcriptional repressors of E-Cadherin, we also investigated their expression in response to emodin treatment. Surprisingly, the results showed that TGF-β1 enhanced the mRNA levels of Snail and Slug by 14.02 and 8.09 fold respectively, when compared with control cells (Fig. 5f,g). Nonetheless, emodin treatment substantially inhibited TGF-β1-induced Snail and Slug transcription in a dose-dependent manner (Snail: 13.6, 24.0, 38.7%, respectively; Slug: 38.0, 54.9, and 73.7%, respectively).

### Emodin suppresses TGF-β1 expression, EMT and fibroblast activation *in vivo*

**Figure 6:**
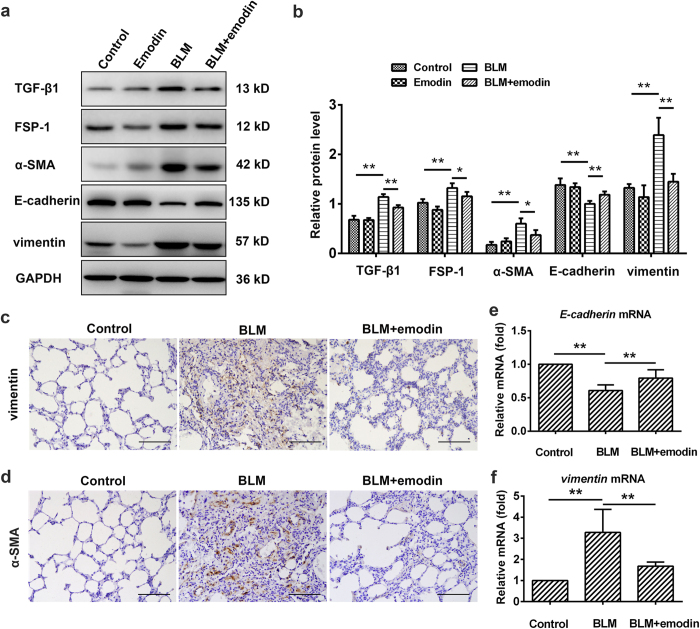
Emodin represses TGF-β1 expression, EMT and fibroblast activation *in vivo*. Rats were intratracheally injected with a single dose of BLM (3.0 mg/kg) and followed by emodin (20 mg/kg) or vehicle by gavage daily for 21 days. (**a**) Western blot was used to analyze the protein levels of TGF-β1, FSP-1, α-SMA, E-cadherin and vimentin in the lung tissues. (**b**) Densitometric analysis of TGF-β1, FSP-1, α-SMA, E-cadherin and vimentin in the immunoblots using GAPDH as the internal reference. (**c,d**) Immunohistochemical staining of vimentin- and α-SMA-positive cells in the lungs, and images at 200× magnification. (**e,f**) Real-time PCR was performed to detect the mRNA levels of E-cadherin and vimentin in the pulmonary tissues derived from each group. Data are expressed as mean ± SD, n = 6, *P < 0.05; **P < 0.01.

We further evaluated the role of emodin in EMT and fibroblast activation during BLM-induced pulmonary fibrosis in rats. Similar to our *in vitro* observations, both RT-PCR and Western blot studies showed that E-cadherin expression was increased and vimentin expression was decreased by emodin treatment (Fig. 6a,b,e,f), providing a critical clue that emodin inhibits EMT *in vivo*. In addition, western blot analysis of lungs showed that the expression of fibroblast-specific protein (FSP)-1 and α-SMA was significantly decreased by emodin treatment (Fig. 6a,b), revealing the inhibitory effect of emodin on fibroblast proliferation and differentiation. Similarly, vimentin and α-SMA-positive cells were both reduced by emodin treatment as determined by immunohistochemical analysis (Fig. 6c,d). All these findings support the notion that emodin inhibits EMT and fibroblast activation in BLM-treated rats. Moreover, we demonstrated that the lungs treated with emodin exhibited a reduced level of TGF-β1, when compared with BLM-treated lungs, which have extensive TGF-β1 (Fig. 6a,b).

### Emodin reduces collagen production and ECM deposition *in vivo*

**Figure 7:**
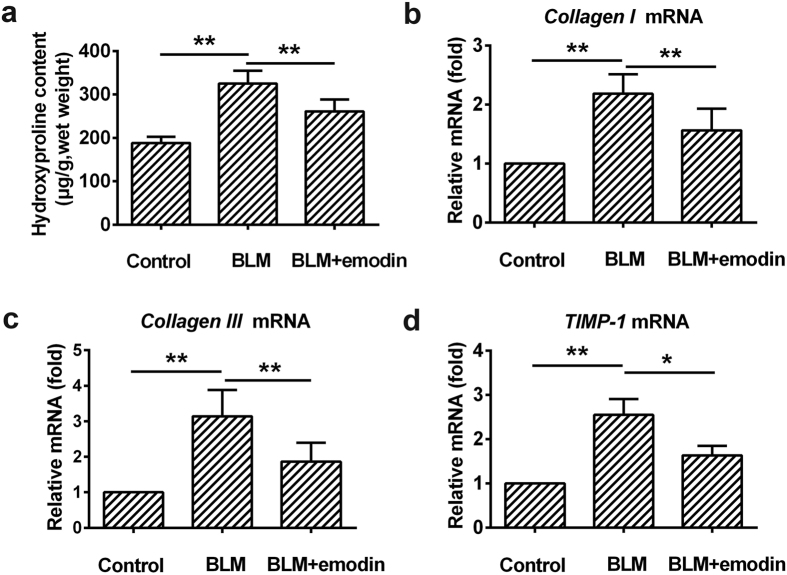
Emodin reduces collagen production and ECM deposition *in vivo*. Rats were intratracheally injected with a single dose of BLM (3.0 mg/kg) and followed by emodin (20 mg/kg) or vehicle by gavage daily for 21 days. (**a**) Hydroxyproline content in the lung tissues. (**b–d**) Real-time PCR was used to detect the mRNA levels of collagen I, collagen III, and TIMP-1 in the lung tissues. Data are expressed as mean ± SD, n = 6, *P < 0.05; **P < 0.01.

The fibroblast and myofibroblast foci secrete excessive ECM. The imbalance between synthesis and degradation of ECM leads to scarring and destruction of lung architecture^5^. We therefore investigated the role of emodin in collagen production by the lungs treated with BLM, which are key contributors of ECM deposition. Our results revealed that BLM treatment profoundly enhanced the levels of hydroxyproline (a main constituent of collagen), collagen I and collagen III in the BLM-treated lungs when compared with control lungs (Fig. 7a–c). Impressively, such changes were significantly ameliorated by emodin treatment, suggesting the anti-fibrotic role of emodin neutralized ECM production and accumulation. Additionally, ECM deposition was also quantified by testing for the level of tissue inhibitors of matrix metalloproteinase (TIMP)-1, which prevents ECM degradation^29^. Here, we observed that BLM stimulation caused increased TIMP-1 level, which was partially reversed by emodin treatment (Fig. 7d), further demonstrated that the inhibitory effects of emodin are partly attributed to increased ECM degradation.

## Discussion

In the present study, we investigated the possible role of emodin in pulmonary fibrosis in *vivo* and *in vitro*. Our data showed that emodin effectively inhibited ECM deposition, lung remodeling, honeycombing and loss of pulmonary function. Emodin significantly induced apoptosis and inhibited migration, proliferation and differentiation of resident lung fibroblasts. In addition, emodin significantly upregulated the epithelial markers and decreased the expression of mesenchymal markers *in vivo* and *in vitro*, suggesting that emodin attenuated BLM-induced pulmonary fibrosis via inhibition of EMT. We also demonstrated that emodin antagonized TGF-β1-mediated canonical Smad signaling and non-canonical MAP kinase (Erk1/2) signaling to downregulate the expression of Slug and Snail, leading to the inhibition of EMT.

IPF is a complex multi-gene and multi-pathway disease with considerable morbidity and mortality^30^. BLM, an antibiotic, is widely used to treat various tumors. Its cytotoxicity occurs primarily in the lung, causing frequent complications, such as pneumonia and pulmonary fibrosis. Similarly, single treatment with BLM induces biochemical and histological changes in rat lungs, simulating human pulmonary fibrosis^31^. Therefore, the BLM-induced rodent model is extensively used to study the mechanism of pulmonary fibrosis. In the current study, we also utilized this model to elucidate the role and underlying mechanisms of emodin in BLM-induced pulmonary fibrosis. Due to the high diagnostic accuracy of CT, IPF is usually diagnosed based on clinical and radiologic features^1^. Interestingly, our data showed that emodin significantly attenuated the honeycombing lesions caused by BLM. Loss of pulmonary function plays a crucial role in IPF. Therefore, improvement in pulmonary function is particularly important. In the present study, we found that emodin significantly improved pulmonary function in animals with pulmonary fibrosis, which is in agreement with a previous study that rhubarb extract containing emodin as the main active ingredient improved pulmonary function in lung cancer patients^32^. Importantly, no side effects were found in our study, indicating the efficacy and safety of emodin, in accordance with earlier preclinical studies, suggesting tolerance, with no reported toxicity^33^,^34^. Based on these observations, we believe that this traditional medicine has a variety of roles other than cathartic effect and may be used as an anti-fibrotic therapy clinically.

IPF is characterized by an abnormal and uncontrolled proliferation of fibroblasts and myofibroblasts, and distortion of lung architecture due to repetitive chronic injury to the AECs followed by disorganized repair process^35^. The poor prognosis of IPF is intimately associated with those increased number of fibroblastic foci, which consist of massive fibroblasts and myofibroblasts^36^. Myofibroblasts that cause exaggerated ECM deposition and contribute to basement membrane damage, epithelial cell death and irreversible fibrotic remodeling, are largely derived from activation and differentiation of resident lung fibroblasts^37^. Talmadge *et al*. (2015) also showed that around 50% of myofibroblasts originate from resident mesenchymal cells. In this study, we found a limited expression of FSP-1 and α-SMA, and less infiltration of α-SMA-positive myofibroblasts following emodin treatment, conforming the inhibitory role of emodin in fibroblast activation, proliferation and differentiation *in vivo*. This is very important for amelioration of pulmonary fibrosis by emodin, supported by another report that ablation of FSP-1 and α-SMA expressions alleviates the course of fibrosis in mouse lung^11^. Local enhancement of active TGF-β1, a critical profibrotic cytokine, mainly produced by AECs in IPF, triggers the differentiation of fibroblasts into myofibroblasts^5^. Hence, we next determined the effect of emodin on TGF-β1-induced myofibroblast differentiation *in vitro*. Impressively, emodin significantly repressed upregulation of α-SMA, a marker of myofibroblast differentiation, in TGF-β1-stimulated HELFs.

In this study, we also investigated the effects of emodin on cellular proliferation, migration and apoptosis in fibroblasts and activated fibroblasts. The results showed that emodin significantly blocked both constitutive and inducible cellular proliferation in fibroblasts, which is consistent with previous studies in hepatocellular carcinoma HepG2 cells where emodin was found to downregulate cyclin D1 expression, a cell cycle regulatory protein^38^. Consistent with this finding, emodin also induced their apoptosis. This is significant because decreased levels of apoptosis in fibroblasts and myofibroblasts are closely linked to the formation of fibrotic lesions and induction of their apoptosis reverses the development of IPF^39^. Further, we observed that caspases mediate the execution phase of apoptosis. Caspases 3, 6 and 7 represent the executioner caspases, in apoptosis. Activated caspase 3 leads to caspase 8 activation, which activates Bax-mediated induction of apoptosis. Our results documented that the expression of cleaved caspase 3, 8 and Bax was upregulated by emodin treatment, clearly indicating that emodin induces caspase-dependent apoptosis in HELFs. This is in agreement with a previous study that emodin induces apoptosis via caspase 3-dependent pathway in HK-2 cells^18^. In addition, anti-apoptotic protein Bcl-2 was significantly decreased, indicating that the downregulation of Bcl-2 expression was likely to be associated with apoptosis induction by emodin in lung fibroblasts and activated fibroblasts. These data indicate that emodin exerts its anti-proliferative activity by inducing apoptosis. However, additional studies are needed to elucidate the effects of emodin on the cell cycle profiles. Furthermore, our data showed that emodin suppressed migration of fibroblasts as well as TGF-β1-stimulated fibroblasts, an aspect that requires further detailed investigation. The above findings further established that emodin ameliorates BLM-induced pulmonary fibrosis by modulating fibroblast/activated fibroblast activities.

In addition to proliferation and differentiation of resident lung fibroblasts, myofibroblasts may also be derived, in part, from EMT. EMT, accounting for about 33% of myofibroblasts, has been implicated in the development of fibrotic diseases^5^,^40^. In this case, TGF-β1 also plays a key role. Thus, studies investigating TGF-β1-induced EMT in human AECs are critical to our understanding of the mechanism of IPF. We found that TGF-β1 triggers the loss of epithelial markers such as E-cadherin in AECs and expression of vimentin and fibronectin, which are specific for mesenchymal cells, consistent with previous studies^41^. Emodin treatment not only inhibited the upregulation of TGF-β1 expression and TGF-β1-induced EMT *in vitro*, but also suppressed TGF-β1 and EMT induced by BLM stimulation *in vivo*, implying that the anti-fibrotic roles of emodin are, at least in part, due to TGF-β1-mediated EMT. Our results are partially in agreement with a recent study by Way *et al*. (2014) who demonstrated that emodin suppresses TWIS-induced EMT in head and neck squamous cell carcinoma cells^19^. It appears that the enhanced expression of E-cadherin by emodin was mediated via suppression of Snail and Slug, endogenous repressors of E-cadherin. TGF-β1 induces EMT by Smad-dependent and Smad-independent pathways^42^. Thus, we hypothesized that emodin exerted its efficacy via inactivation of key molecules in the TGF-β1 signaling pathway. We found that the phosphorylation of Smad2, Smad3 and Erk1/2 was significant repressed by emodin in TGF-β1-primed A549 cells. This finding was validated in BLM-induced pulmonary fibrosis in rats, evidenced by decreased level of phosphorylation of Smad2, Smad3 and Erk1/2 in emodin-treated groups (data not shown here). Hence, we speculated that emodin may play a hidden therapeutic role in pulmonary fibrosis by blocking TGF-β1 signaling or inhibiting EMT.

Injured Type II alveolar epithelial cells repeatedly release a variety of mediators, cytokines and growth factors, which promote excessive activation of fibroblasts, EMT and ECM production, thereby leading to IPF^43^,^44^. TIMP-1, a prototypic and original ancestral member of the TIMP family, is primarily expressed on alveolar epithelial cells and macrophages, and upregulated during pulmonary fibrosis^45^. Its expression is associated with the stages of disease progression, via preventing MMP-induced ECM degradation and, as a consequent, participating in ECM accumulation^46^. In our present study, we demonstrated that emodin significantly inhibited TIMP-1 expression, which is consistent with previous observation on the activity of emodin in rat hepatic stellate cells^47^. Emodin treatment also reduced the level of collagens, the primary components of ECM, indicating that emodin treatment significantly maintained the balance between the synthesis and degradation of ECM components in the local lung microenvironment, which is important for the amelioration of pulmonary fibrosis.

In summary, our results indicate that emodin inhibits TGF-β1 signaling pathway and subsequently suppresses fibroblast activation, EMT and eventual ECM deposition, thus leading to an improvement in BLM-induced pulmonary fibrosis and pulmonary function.

## Methods

### Reagents and antibodies

Recombinant human TGF-β1 was purchased from R&D Systems (Minneapolis, MN, USA), emodin was obtained from Shanghai future industry Limited by Share Ltd (Shanghai, China) and BLM was acquired from Nippon Kayaku (Tokyo, Japan). The primary antibodies described in the study include: anti-E-cadherin, anti-vimentin, anti-cleaved caspase-3, anti-phospho-Smad2, anti-phospho-Smad3, anti-Smad2, anti-Smad3, anti-phospho-Erk1/2 and anti-Erk1/2 (Cell Signaling Technology, CA, USA); anti-caspase-3, anti-Bax (Santa Cruz Biotechnology, CA, USA); anti-fibronectin (Proteintech, Chicago, USA); anti-caspase-8, anti-Bcl-2 (Absci, MD, USA); anti-TGF-β1, anti-FSP-1, anti-α-SMA (Abcam, USA); and anti-GAPDH (Beyotime Institute of Biotechnology, Haimen, China). Other reagents were obtained from Beyotime Institute of Biotechnology unless otherwise indicated.

### Animals

Adult male Sprague-Dawley rats, each weighing 180–220 g, were purchased from the Experimental Animal Center of Chinese Academy of Sciences in Shanghai. All the animals were acclimatized in a room (12/12 h light/dark cycle; 25 ± 2 °C) and allowed free access to diet and water ad libitum. All the animal experiments were approved by the Animal Care and Use Committee of Shanghai Medical College, Fudan University, and were performed in strict accordance with approved guidelines.

### Animal treatment

All the animals were randomly divided into one of the four groups: (1) intratracheal saline plus 0.5% sodium carboxymethyl cellulose (0.5% CMC-Na) intragastrically (control group); (2) intratracheal saline plus 20 mg/kg of emodin in 0.5% CMC-Na i.g. (emodin group); (3) intratracheal BLM plus 0.5% CMC-Na i.g. (BLM group); and (4) intratracheal BLM plus 20 mg/kg emodin in 0.5% CMC-Na i.g. (BLM+emodin group). BLM (3.0 mg/kg; Nippon Kayaku, Tokyo, Japan) in saline or saline alone was administered intratracheally to the rats on day 0. One day after BLM challenge, emodin or 0.5% CMC-Na was administered orally daily for 21 days. Immediately after all the rats were euthanized, lungs were harvested for the following experiments.

### Quantum GX micro-CT Imaging system

Rats were anesthetized by isoflurane and transferred into a PerkinElmer Quantum Gx apparatus (PerkinElmer, USA) to scan the area of the lung fields. The scanned images were uploaded as BMP files into the Proscanarray express software (PerkinElmer, USA) and analyzed with Analyze 12.0.

### Evaluation of pulmonary function

Rats were anesthetized with 6% chloral hydrate in saline (600 mg/kg i.p.) and transferred into a plethysmographic chamber (4665 ml volume) to determine the pulmonary function using the Anires2005 system (Beijing Biolab, Beijing, China). This system automatically calculates and displays pulmonary parameters including dynamic compliance (Cdyn), inspiratory resistance (Ri) and expiratory resistance (Re) on the basis of changes detected.

### Histopathological examination

Left lungs were fixed in 4% paraformaldehyde in 0.1 M PBS overnight and embedded in paraffin. Then lung sections (5 μm) were prepared and stained with Masson’s trichrome staining. Slices were analyzed under a microscope (Leica DM 4000M, Germany).

### Hydroxyproline content

The hydroxyproline content in the lungs was determined using a kit (Nanjing Jiancheng Bioengineering Institute, Nanjing, China) according to the manufacturer’s instructions.

### Immunohistochemistry

Tissue sections were deparaffinized and rehydrated using a graded ethanol series. After antigen retrieval, eliminating endogenous peroxidase and pre-incubating with 5% BSA to block background staining, sections were incubated with rabbit anti-α-SMA antibody (1:100) or rabbit anti-vimentin antibody (1:100) at 4 °C overnight. The color reaction was then made with HRP-linked polymer detection system and counterstained with hematoxylin.

### Cell preparation and culture

Human alveolar epithelial A549 cells and human embryo lung fibroblasts (HELFs) were purchased from FuDan IBS Cell Center (Shanghai, China), and cultured in Dulbecco’s modified Eagle’s medium (DMEM, 4.5 g/L glucose) (Life Technologies, USA) supplemented with 10% fetal bovine serum and antibiotics (100 KU/L penicillin and 100 mg/L streptomycin) in an incubator at 37 °C with 5% CO_2_ atmosphere.

### Morphological analysish

A549 cells were treated with culture medium alone (control) or with 15, 30, and 60 μM of emodin for 24 and 48 h, in the presence and absence of TGF-β1 (10 ng/mL). Cell morphology was observed under an inverted phase-contrast microscope (Leica, Germany). Digital images of cells were randomly captured and examined for morphologic characteristics consistent with EMT at 400x magnification.

### Immunofluorescence staining

A549 cells were seeded on glass slides and treated as indicated for 24 h. The slides were washed with PBS, followed by fixation with 4% paraformaldehyde for 10 min and permeabilization with 0.1% Triton X-100 in PBS. The cells were blocked with 2% BSA at room temperature for 1 h and incubated with primary antibodies (E-cadherin, 1:200; fibronectin, 1:200; α-SMA, 1:200) at 4 °C overnight. After incubation, samples were washed three times with PBS and incubated with corresponding secondary antibodies (1:200, Beyotime Institute of Biotechnology, Haimen, China). The nuclei were stained with DAPI. Samples were washed three times with PBS, coverslips mounted in 90% glycerol in PBS, and fluorescence detected using a Zeiss LSM 710 confocal laser system.

### Cell viability analysis

HELFs were seeded in a 96-well plate and treated with different doses of emodin in the presence and absence of TGF-β1 (10 ng/mL) as indicated. Cell viability was determined according to the manufacturer’s instructions of Cell Counting Kit-8 (CCK-8) Kit (Dojindo, Japan).

### Wound healing assay

HELFs were seeded in a six-well plate and treated with emodin in the presence and absence of TGF-β1 (10 ng/mL) for the indicated period. Three parallel lines were drawn on the underside of each well to demarcate the wound areas for analysis. Before inflicting the wound, the cells were fully confluent. A scratch was made in the center of the culture well using a sterile 200 μL micropipette tip. The wounds were observed using bright field microscopy (Olympus, Tokyo, Japan) and multiple images were obtained at areas flanking the intersections of the wound and the marker lines after the scratch at 0 h, 24 h, and 48 h. Images were obtained for analysis using Image J software.

### Apoptotic cell determination

HELFs were plated in a six-well plate (2 × 10^5^ cells/well) and treated with emodin in the presence and absence of TGF-β1 (10 ng/mL) for 24 h. The cellular apoptotic rate was determined using Annexin V, FITC Apoptosis Detection Kit (Dojindo, Japan).

### Quantitative real-time PCR

Total RNA was extracted from lung tissues and cells using TRIxzol Reagent (Invitrogen Corporation, CA, USA). First-strand cDNA was synthesized and amplified from 0.5 μg of total RNA using the ReverTra Ace qPCR RT Kit (Toyobo, Tokyo, Japan). Then the mRNA levels of collagen I, collagen III, TIMP-1, E-cadherin, vimentin, TGF-β1, Snail and Slug were analyzed by quantitative real-time PCR (iCyler iQ Real-time PCR Detection System, Bio-Rad Laboratories Inc., USA) using SYBR Green Real-time PCR Master Mix (Toyobo, Japan) in a total volume of 20 μL. Relative levels of mRNA expression were normalized to GAPDH expression for each gene. Primers are listed in Table 1.

### Western blot

The samples of lung tissues and cells were homogenized in RIPA lysis buffer with protease or phosphatase inhibitor (Roche) and then centrifuged (12000 rpm, 15 min, 4 °C) to obtain supernatants. The total protein concentration was measured by bicinchoninic acid (BCA). Total protein (10–50 μg) was loaded into each lane onto SDS-polyacrylamide gels and transferred to PVDF membranes (Millipore, MA, USA) and incubated with appropriate primary antibodies overnight at 4 °C. After reacting with HRP-labeled secondary antibodies, the membranes were probed using an ECL-Plus detection kit probed (Tiangen Biotech Co. Ltd., Beijing, China) and then scanned with Imagequant LAS4000 mini (GE Healthcare Life Sciences, CT, USA). The results were analyzed by a densitometry system named Image J.

### Data analysis

Data are expressed as mean ± standard deviation (SD), statistical analysis was performed using Students t-test or One-way analysis of variance (ANOVA). A value of P < 0.05 was considered statistically significant.

## Additional Information

**How to cite this article**: Guan, R. *et al*. Emodin ameliorates bleomycin-induced pulmonary fibrosis in rats by suppressing epithelial-mesenchymal transition and fibroblast activation. *Sci. Rep.* **6**, 35696; doi: 10.1038/srep35696 (2016).

## Figures and Tables

**Table 1:** Primer sets used for real-time PCR.

Gene	Sense Primer(5′-3′)	Antisense Primer(5′-3′)
rat TIMP-1	GACCACCTTATACCAGCGTT	GTCACTCTCCAGTTTGCAAG
rat collagen I	ATCCTGCCGATGTCGCTAT	CCACAAGCGTGCTGTAGGT
rat collagen III	CTGGTCCTGTTGGTCCATCT	ACCTTTGTCACCTCGTGGAC
rat E-cadherin	GGCCCAGGAGCTGACAAAC	CCAGAGGCTGCGTCACTTTC
rat vimentin	ATGAAAGTGTGGCTGCCAAGAAC	GTGACTGCACCTGTCTCCGGTA
human TGF-β1	AAGGACCTCGGCTGGAAGTG	CCGGGTTATGCTGGTTGTA
human Snail	GCCTAGCGAGTGGTTCTTCT	TAGGGCTGCTGGAAGGTAAA
human Slug	GAGCATTTGCAGACAGGTCA	ACAGCAGCCAGATTCCTCAT
rat GAPDH	AAGGTGGTGAAGCAGGCGGC	GAGCAATGCCAGCCCCAGCA
human GAPDH	TGACTTCAACAGCGACACCCA	CACCCTGTTGCTGTAGCCAAA

## References

[b1] Raghu G. . An official ATS/ERS/JRS/ALAT statement: idiopathic pulmonary fibrosis: evidence-based guidelines for diagnosis and management. Am J Respir Crit Care Med. 183, 788–824 (2011). 21471066 10.1164/rccm.2009-040GL PMC5450933

[b2] King T. J. . A phase 3 trial of pirfenidone in patients with idiopathic pulmonary fibrosis. N Engl J Med. 370, 2083–2092 (2014). 24836312 10.1056/NEJMoa1402582

[b3] Xaubet A., Serrano-Mollar A. & Ancochea J. Pirfenidone for the treatment of idiopathic pulmonary fibrosis. Expert Opin Pharmacother. 15, 275–281 (2014). 24308635 10.1517/14656566.2014.867328

[b4] Karimi-Shah B. A. & Chowdhury B. A. Forced vital capacity in idiopathic pulmonary fibrosis–FDA review of pirfenidone and nintedanib. N Engl J Med. 372, 1189–1191 (2015). 25806913 10.1056/NEJMp1500526

[b5] King T. J., Pardo A. & Selman M. Idiopathic pulmonary fibrosis. Lancet. 378, 1949–1961 (2011). 21719092 10.1016/S0140-6736(11)60052-4

[b6] Bonella F., Stowasser S. & Wollin L. Idiopathic pulmonary fibrosis: current treatment options and critical appraisal of nintedanib. Drug Des Devel Ther. 9, 6407–6419 (2015). 10.2147/DDDT.S76648 PMC4686227 26715838

[b7] Willis B. C. . Induction of epithelial-mesenchymal transition in alveolar epithelial cells by transforming growth factor-beta1: potential role in idiopathic pulmonary fibrosis. AM J Pathol. 166, 1321–1332 (2005). 15855634 10.1016/s0002-9440(10)62351-6 PMC1606388

[b8] Olson N. & van der Vliet A. Interactions between nitric oxide and hypoxia-inducible factor signaling pathways in inflammatory disease. Nitric Oxide 25, 125–137 (2011). 21199675 10.1016/j.niox.2010.12.010 PMC3090692

[b9] Milara J. . Sphingosine-1-phosphate is increased in patients with idiopathic pulmonary fibrosis and mediates epithelial to mesenchymal transition. Thorax 67, 147–156 (2012). 22106015 10.1136/thoraxjnl-2011-200026

[b10] Willis B. C. & Borok Z. TGF-beta-induced EMT: mechanisms and implications for fibrotic lung disease. Am J Physiol Lung Cell Mol Physiol. 293, L525–L534 (2007). 17631612 10.1152/ajplung.00163.2007

[b11] Chen Y. L. . Sorafenib ameliorates bleomycin-induced pulmonary fibrosis: potential roles in the inhibition of epithelial-mesenchymal transition and fibroblast activation. Cell Death Dis. 4, e665 (2013). 23764846 10.1038/cddis.2013.154 PMC3698540

[b12] Khalil N. . Increased production and immunohistochemical localization of transforming growth factor-beta in idiopathic pulmonary fibrosis. Am J Respir Cell Mol Biol. 5, 155–162 (1991). 1892646 10.1165/ajrcmb/5.2.155

[b13] Hinz B. . The myofibroblast: one function, multiple origins. Am J Pathol. 170, 1807–1816 (2007). 17525249 10.2353/ajpath.2007.070112 PMC1899462

[b14] Nieto M. A. The snail superfamily of zinc-finger transcription factors. Nat Rev Mol Cell Biol. 3, 155–166 (2002). 11994736 10.1038/nrm757

[b15] Takemasa A., Ishii Y. & Fukuda T. A neutrophil elastase inhibitor prevents bleomycin-induced pulmonary fibrosis in mice. Eur Respir J. 40, 1475–1482 (2012). 22441751 10.1183/09031936.00127011

[b16] Xu J. D. . Emodin induces chloride secretion in rat distal colon through activation of mast cells and enteric neurons. Br J Pharmacol. 165, 197–207 (2012). 21718311 10.1111/j.1476-5381.2011.01573.x PMC3252977

[b17] Chen T. . Emodin ameliorates high glucose induced-podocyte epithelial-mesenchymal transition *in-vitro* and *in-vivo*. Cell Physiol Biochem. 35, 1425–1436 (2015). 25791065 10.1159/000373963

[b18] Wang C. . Emodin induces apoptosis through caspase 3-dependent pathway in HK-2 cells. Toxicology. 231, 120–128 (2007). 17240509 10.1016/j.tox.2006.11.064

[b19] Way T. D. . Emodin represses TWIST1-induced epithelial-mesenchymal transitions in head and neck squamous cell carcinoma cells by inhibiting the beta-catenin and Akt pathways. Eur J Cancer 50, 366–378 (2014). 24157255 10.1016/j.ejca.2013.09.025

[b20] Wang C. H. . Effect of emodin on pancreatic fibrosis in rats. World J Gastroenterol 13, 378–382 (2007). 17230605 10.3748/wjg.v13.i3.378 PMC4065891

[b21] Zhan Y. . Emodin on hepatic fibrosis in rats. Chin Med J (Engl). 113, 599–601 (2000). 11776026

[b22] Chen X. H. . Inhibitory effect of emodin on bleomycin-induced pulmonary fibrosis in mice. Clin Exp Pharmacol Physiol. 36, 146–153 (2009). 18785980 10.1111/j.1440-1681.2008.05048.x

[b23] Gao L. . Glycyrrhizic acid alleviates bleomycin-induced pulmonary fibrosis in rats. Front Pharmacol. 6, 215 (2015). 26483688 10.3389/fphar.2015.00215 PMC4589765

[b24] Lorena D., Uchio K., Costa A. M. & Desmouliere A. Normal scarring: importance of myofibroblasts. Wound Repair Regen. 10, 86–92 (2002). 12028520 10.1046/j.1524-475x.2002.00201.x

[b25] Baum B., Settleman J. & Quinlan M. P. Transitions between epithelial and mesenchymal states in development and disease. Semin Cell Dev Biol. 19, 294–308 (2008). 18343170 10.1016/j.semcdb.2008.02.001

[b26] Zavadil J. & Bottinger E. P. TGF-beta and epithelial-to-mesenchymal transitions. Oncogene. 24, 5764–5774 (2005). 16123809 10.1038/sj.onc.1208927

[b27] Zhou G. . Hypoxia-induced alveolar epithelial-mesenchymal transition requires mitochondrial ROS and hypoxia-inducible factor 1. Am J Physiol Lung Cell Mol Physiol. 297, L1120–L1130 (2009). 19801454 10.1152/ajplung.00007.2009 PMC2793183

[b28] Massague J. & Chen Y. G. Controlling TGF-beta signaling. Genes Dev. 14, 627–644 (2000). 10733523

[b29] Wang B. L. . Unbalanced MMP/TIMP-1 expression during the development of experimental pulmonary fibrosis with acute paraquat poisoning. Mol Med Rep. 4, 243–248 (2011). 21468558 10.3892/mmr.2011.425

[b30] Crystal R. G. . Future research directions in idiopathic pulmonary fibrosis: summary of a National Heart, Lung, and Blood Institute working group. Am J Respir Crit Care Med. 166, 236–246 (2002). 12119236 10.1164/rccm.2201069

[b31] Song N. . Vagotomy attenuates bleomycin-induced pulmonary fibrosis in mice. Sci Rep. 5, 13419 (2015). 26289670 10.1038/srep13419 PMC4542162

[b32] Yu H. M., Liu Y. F., Cheng Y. F., Hu L. K. & Hou M. Effects of rhubarb extract on radiation induced lung toxicity via decreasing transforming growth factor-beta-1 and interleukin-6 in lung cancer patients treated with radiotherapy. Lung Cancer 59, 219–226 (2008). 17870203 10.1016/j.lungcan.2007.08.007

[b33] Xu X. C. & Lin S. Z. [Experimental advance of applying emodin for prevention and treatment of liver diseases]. Zhongguo Zhong Xi Yi Jie He Za Zhi. 28, 91–93 (2008). 18418981

[b34] Liu A. . Emodin potentiates the antitumor effects of gemcitabine in pancreatic cancer cells via inhibition of nuclear factor-kappaB. Mol Med Rep. 4, 221–227 (2011). 21468555 10.3892/mmr.2011.414

[b35] Wolters P. J., Collard H. R. & Jones K. D. Pathogenesis of idiopathic pulmonary fibrosis. Annu Rev Pathol. 9, 157–179 (2014). 24050627 10.1146/annurev-pathol-012513-104706 PMC4116429

[b36] Pechkovsky D. V. . STAT3-mediated signaling dysregulates lung fibroblast-myofibroblast activation and differentiation in UIP/IPF. Am J Pathol. 180, 1398–1412 (2012). 22322297 10.1016/j.ajpath.2011.12.022

[b37] Hinz B. . Recent developments in myofibroblast biology: paradigms for connective tissue remodeling. Am J Pathol. 180, 1340–1355 (2012). 22387320 10.1016/j.ajpath.2012.02.004 PMC3640252

[b38] Subramaniam A. . Emodin inhibits growth and induces apoptosis in an orthotopic hepatocellular carcinoma model by blocking activation of STAT3. Br J Pharmacol. 170, 807–821 (2013). 23848338 10.1111/bph.12302 PMC3799595

[b39] Fattman C. L. Apoptosis in pulmonary fibrosis: too much or not enough? Antioxid Redox Signal 10, 379–385 (2008). 18031201 10.1089/ars.2007.1907

[b40] Ikegami T., Zhang Y. & Matsuzaki Y. Liver fibrosis: possible involvement of EMT. Cells Tissues Organs 185, 213–221 (2007). 17587827 10.1159/000101322

[b41] Phan S. H. Genesis of the myofibroblast in lung injury and fibrosis. Proc Am Thorac Soc. 9, 148–152 (2012). 22802289 10.1513/pats.201201-011AW PMC5830705

[b42] Zhang Y. E. Non-Smad pathways in TGF-beta signaling. Cell Res. 19, 128–139 (2009). 19114990 10.1038/cr.2008.328 PMC2635127

[b43] Vancheri C., Failla M., Crimi N. & Raghu G. Idiopathic pulmonary fibrosis: a disease with similarities and links to cancer biology. Eur Respir J. 35, 496–504 (2010). 20190329 10.1183/09031936.00077309

[b44] Selman M. & Pardo A. Stochastic age-related epigenetic drift in the pathogenesis of idiopathic pulmonary fibrosis. Am J Respir Crit Care Med. 190, 1328–1330 (2014). 25496096 10.1164/rccm.201411-1953ED

[b45] Handsley M. M. & Edwards D. R. Metalloproteinases and their inhibitors in tumor angiogenesis. Int J Cancer. 115, 849–860 (2005). 15729716 10.1002/ijc.20945

[b46] Ohbayashi H. Matrix metalloproteinases in lung diseases. Curr Protein Pept Sci. 3, 409–421 (2002). 12370004 10.2174/1389203023380549

[b47] Gui M. . Inhibitory effect of emodin on tissue inhibitor of metalloproteinases-1 (TIMP-1) expression in rat hepatic stellate cells. Dig Dis Sci. 52, 200–207 (2007). 17160480 10.1007/s10620-006-9321-z

